# A Comparative Analysis of Cell Proliferation and Wound Closure in Cultured Gingival Epithelial Cells Using Plasma Rich in Growth Factors and Platelet-Rich Plasma Containing Leukocytes

**DOI:** 10.1055/s-0044-1801274

**Published:** 2025-01-20

**Authors:** Yuri Watanabe, Katsumitsu Shimada, Yousuke Doi, Takuyoshi Higuchi, Yoshiya Kato, Xianqi Li, Yuji Kurihara, Satoshi Murakami

**Affiliations:** 1 Hard Tissue Pathology Unit, Graduate School of Oral Medicine, Matsumoto Dental University, Nagano, Japan; 2 Department of Oral and Maxillofacial Surgery, Matsumoto Dental University, Nagano, Japan; 3 Department of Clinical Pathophysiology, School of Dentistry, Matsumoto Dental University, Nagano, Japan; 4 Department of Orthodontic Clinic, Matsumoto Dental University Hospital, Nagano, Japan

**Keywords:** PRGF, PRP, gingival epithelial cells, proliferation, wound closure

## Abstract

**Objectives:**

Plasma rich in growth factors (PRGF) is presumed to be able to stimulate the regeneration of skin and periodontal tissue. This effect can be attributed to the fact that PRGF contains fewer leukocyte-derived interleukins in comparison to platelet-rich plasma (PRP). However, a comparison of the effects of PRGF and PRP on gingival epithelial cells has not been conducted yet. Therefore, our objective was to clarify and compare the effects of PRGF and PRP on gingival epithelial cell proliferation, wound healing, and gene expression.

**Materials and Methods:**

PRGF and PRP were obtained from three donors. A complete medium containing bovine pituitary extract (BPE) and growth factors was used as a positive control (PC), while a medium without BPE was used as a negative control (NC). We evaluated the presence of platelets and leukocytes, as well as the number of leukocytes, in PRP and PRGF using the cell block method and a cell counting chamber. We assessed gingival epithelial cell proliferation with WST-1 and wound healing by using cell-free culture inserts. To examine the mRNA expression of tumor necrosis factor-α (TNF-α), which is related to cell growth inhibition, and integrin β4, which contributes to cell adhesion, we used quantitative reverse transcription polymerase chain reactions (RT-PCRs) under PRGF and PRP samples in vitro. The nonparametric data were analyzed using the Kruskal–Wallis test.

**Results:**

Large quantities of platelets were observed in both PRGF and PRP. The leukocyte concentration in PRGF was generally lower than that in PRP. Our report indicated that cell proliferation was significantly higher in PRGF than in PRP on day 1 and 2. We found that there was no significant difference in the wound closure rate between PRGF and PRP in comparison to their respective control groups. The quantitative RT-PCR revealed insignificant differences in mRNA expression as TNF-α and integrin β4 between PRGF and PRP in comparison to the each of their respective control groups.

**Conclusion:**

Our research indicated that PRGF can promote the proliferation of gingival epithelium more than PRP, contributing to the healing of periodontal tissue. TNF-α and integrin β4 mRNA expression may not be significantly involved in wound closure within the gingival epithelium under the influence of PRGF and PRP.

## Introduction

The surface of a wound is covered with blood components such as blood clots, blood cells, and fibrin. The wound begins to refill with granulation tissue. Epithelial cells migrate into the blood components and granulation tissue, covering the wound surface and maturing for differentiation. 1 For a wound to heal, it is important for the epithelial barrier to protect it from infection. 2 To protect against infection, claudins regulate the gate function as paracellular tight-junction channels. 3 Additionally, antimicrobial peptides such as β-defensin limit bacterial invasion. 4 Thus, an epithelial barrier separates the gingival connective tissue from the external environment and protects it from bacteria. 5 To establish the epithelial barrier, oral mucosal cells must proliferate and migrate toward the wound area to promote closure. 6 Regenerative therapy for promoting the wound healing process consists of stem cell implantation, scaffold construction, and signal transduction. 7 Signal transduction methods are useful for mediating growth factors such as epidermal growth factor (EGF), platelet-derived growth factor (PDGF), and insulin-like growth factor (IGF). 8 However, the need to target various kinds of cells in wound healing demands a balanced combination of mediators. This could be explained by the fact that a mixture of growth factors would be more effective than a single puriﬁed molecule. 9 Based on this, platelet-rich plasmas (PRPs) are developed by centrifuging whole blood to concentrate various growth factors released from platelets. 10 These varied growth factors promote tissue regeneration and wound healing. Therefore, PRP is applied to pressure ulcers 11 and burns of the skin. 12 Derivatives of PRPs include formulations such as leukocyte-platelet–rich plasma (L-PRP), platelet-rich fibrin (PRF), 13 and plasma rich in growth factors (PRGF). 14 Because the platelet concentration in PRP is higher, it may possibly become contaminated with leukocytes during the production process. 15 Choukroun has developed PRF that is collected without any anticoagulants and is immediately centrifuged. 13 PRF extracts contain higher levels of platelets and PDGFs such as PDGF, transforming growth factor-β (TGF-β), and vascular endothelial growth factor (VEGF). 15 Furthermore, the modified PRF was developed as leucocyte-poor PRF. The main difference is that only very low amounts of leucocytes are collected owing to the specific separator gel. 16 These have attracted attention due to their ease of preparation, clinical efficacy, and ability to eliminate proinflammatory factors.

An improved system for producing PRP has been developed by the Biotechnology Institute (BTI, Basque, Spain). 17 In recent years, a system called ENDORET PRGF has been approved for bioadaptation in Japan. This system is said to enable clearer fraction separation of platelets, leukocytes, and erythrocytes than the conventional L-PRP method. The system makes it possible to obtain PRGF by excluding the fraction of leukocytes. 18 Previous research has shown that in comparison to PRP, PRGF contains several significantly poor subtypes of interleukin released from leukocytes. 15 19 Furthermore, it is conceivable that PRGF reduces inflammatory microenvironments by altering the secretion of interleukin, which is induced to promote tissue regeneration. 20 In recent years, PRGF has been attracting attention for the regeneration of not only the alveolar bone but also periodontal tissue, especially in dental clinical practice. Several in vitro studies have recently demonstrated that PRGF promotes the proliferation of the cells that compose periodontal tissue, including gingival fibroblasts, alveolar osteoblasts, and vessel endothelial cells. 21 22

In this study, we targeted L-PRP and PRGF as platelet concentrates. The reason for this is to compare PRGF—a multiplatelet plasma derived from L-PRP, minus the leukocyte fraction—with L-PRP to elucidate the differences in the effects of leukocyte-derived factors on oral cells. However, due to the lack of research on the oral epithelium, no consensus has been reached on how PRGF promotes or inhibits proliferation and migration in vitro, especially in comparison to PRP.

We hypothesize that PRGF (rather than PRP) promotes the proliferation and migration of gingival epithelial cells. To test this hypothesis, we compared how the use of PRGF and PRP affected cell proliferation, wound healing, and the expressions of genes that can be attributable to cell proliferation and migration.

## Materials and Methods

### Collection and Preparation of PRGF and PRP

This study was approved by the Ethics Committee of Matsumoto Dental University (approval number 0363) and conducted in accordance with the principles of the Declaration of Helsinki (version 2013). After receiving their informed written consent, blood was collected from healthy donors into 9-mL tubes with 3.8% (wt/v) sodium citrate. The donors were male, aged 28, 30, and 38 years, respectively. None of the donors had any systemic diseases (▶ Table 1 ). The evaluation of blood components, cell proliferation, and the quantitative reverse transcription polymerase chain reactions (RT-PCR) was performed on samples from three donors, whereas the wound healing assay was conducted on the sample from one donor (a 38-year-old male). Blood was centrifuged at 580 g for 8 minutes at room temperature (Endoret System, BTI Biotechnology Institute, S.L., Spain) to obtain PRGF and PRP. To prepare PRGF, the whole plasma column, except the layer that contains leukocytes, was collected from one-third of the tubes of each donor. To obtain PRP, the last 1 mL of plasma (including the buffy coat) was gathered from the rest of the tubes, in addition to the leukocyte layer over the red fraction. The obtained PRGF and PRP were incubated at 37°C for 1 hour (Hybridization Incubator HB-80, Taitec, Saitama, Japan). The incubated PRGF and PRP were centrifuged at 1000 g for 10 minutes at room temperature. Supinates of PRGF and PRP were dispensed to store at −80°C before adding the medium for all assays.

**Table 1:** Leukocyte count of PRGF and PRP

Age (y old)	Gender	PRGF (×10 ^6^ /mL)	PRP (×10 ^6^ /mL)
28	Male	0.3	5.0
30	Male	0.5	6.0
38	Male	0.2	6.2
Median		0.3	6.0

Abbreviations: PRGF, plasma rich in growth factors; PRP, platelet-rich plasma.

### Cell Block Preparation Method

The cell block preparation method was applied to the O.T.C. compound (Sakura Finetech Japan, Tokyo, Japan). 23 The O.T.C compound consists of 10.24% polyvinyl alcohol, 4.26% polyethylene glycol, and an inert substance composed mainly of 85.50% water. It is primarily used as an embedding medium for frozen tissue sections. After centrifuging whole blood, these cellular fractions of PRGF and PRP were collected and fixed in 99.5% ethanol (Wako/FUJIFILM, Osaka, Japan) for 30 minutes. The ethanol was discarded and left for 1 minute. The O.C.T. compound was added dropwise and stirred. Subsequently, 99.5% ethanol was added and left until the O.C.T. compound hardened. The cells hardened with O.C.T compound were then placed in a tissue embedding cassette (Murazumi, Osaka, Japan). The embedded cells were subjected to Paraffin infiltration for 14 hours (ETP, Sakura Finetech Japan, Tokyo, Japan). After the paraffin blocks were processed, 3-µm specimens were sectioned and stained with conventional Hematoxylin-Eosin to observe the morphology of the cells, followed by a Giemsa staining to identify the platelet.

### Microscopic Cell Count

Leukocytes were counted using Türk's solution and counting chambers. 24 As part of the examination, 18 or 20 µL of Türk's solution (Sigma-Aldrich, Saint Louis, Missouri) were mixed with 2 µL of the PRP or 20 µL PRGF sample, respectively, to stain the leukocytes. After 1 minute of staining, these mixtures of PRGF or PRP were transferred to a Burker-Turk counting chamber (Erma Inc., Saitama, Japan) or Fuchs-Rosenthal counting chamber (Sunlead Glass Corp., Saitama, Japan). All visible PRGF and PRP leukocytes in the four squares of the counting chamber were counted via microscopic observation using 40-fold and 100-fold magnification, respectively.

### Cell and Cell Culture

Normal human gingival epithelium progenitors, pooled (HGEPp; CELLnTEC Advanced Cell Systems, Bern, Switzerland) were cultured in Cnt-57 medium (CELLnTEC Advanced Cell Systems, Bern, Switzerland).

A BPE-containing medium was used as the PC, and a BPE-free medium was used as the NC. The medium was supplemented with 15% PRGF or 15% PRP at 37°C in 5% CO _2_ .

### Cell Proliferation

HGEPp were seeded in 96-well culture plates at a density of 1,000 cells/well (2,857 cells/cm ^2^ ) and maintained in a cell incubator at 37°C and 5% CO _2_ for 24 hours. The culture medium was then replaced with PC, NC, PRGF, and PRP. Cell proliferation after 1, 2, 3, 7, and 14 days was quantified using the WST-1 (tetrazolium salt, 4-[3-(4-iodophenyl)-2-(4-nitrophenyl)-2H-5-tetrazolio]-1,3-benzene disulfonate) colorimetric assay (Sigma-Aldrich, Saint Louis, Missouri). At each interval, wells were incubated with WST-1 reagent at 37°C for 1 hour, following with the manufacturer's instructions. Absorbance at 450/620 nm is directly proportional to the number of living cells. Background values were subtracted from the sample values.

### Wound Healing Assay

To quantify the wound healing potential of PRGF and PRP, HGEPp were plated in culture inserts (NUNC, Thermo Fisher Scientific Inc., Massachusetts) placed on a 24-well plate at high density and grown until confluence. After the inserts were carefully removed, two separated cell monolayers with a cell-free gap of ∼500 µm thickness were created. The cells were washed with Phosphate-buffered saline and incubated in triplicate with PC, NC, PRGF, and PRP for 8, 12, 14, 16, 18, 20, 22, and 24 hours. To quantify the area of wound healing, phase contrast images of the central part of the septum before treatment and after each treatment interval were captured with an all-in-one fluorescence microscope (BZ-X710, Keyence, Osaka, Japan). The gap area was measured at 8, 12, 14, 16, 18, 20, 22, and 24 hours from initial treatment using ImageJ Software (NIH, Bethesda, Maryland). The wound closure rate was defined as: (area of intercellular gap immediately after removing the insert − area of the intercellular gap at each time point)/(area of intercellular gap immediately after removing the insert).

### Real-Time RT-PCR

To compare transcription levels of a panel of genes in the differently treated HGEPp, a real-time RT-PCR was performed. Thus, HGEPp were grown for 24 hours with PC, NC, PRGF, and PRP. Total RNA was extracted as per the manufacturer's protocol (Direct-zol RNA Microprep, Zymo Research, Irvine, California), and treated with TRIzol Reagent (Invitrogen, Darmstadt, Germany) for 15 minutes at room temperature. Total RNA was stored at −80°C until use. For each sample, cDNA was synthesized from 250 ng total RNA using the SuperScript IV Reverse Transcriptase (Invitrogen, Vilnius, Lithuania). Real-time PCR assays were performed with a Thermal Cycler Dice Real-Time System II (Takara Bio Inc., Shiga, Japan). The reaction mixture contained 1 µL cDNA from the RT reaction, together with forward and reverse specific primers (10 µM each) and TB Green Premix Ex Taq II (Takara Bio Inc., Shiga, Japan) in a final reaction volume of 25 µL. The thermal cycling conditions were as follows: an initial polymerase activating step at 95°C for 3 minutes (denaturation step), followed by 40 cycles, 30 seconds at 60°C (annealing step/extension step), during which data were collected. Each assay included a NC consisting of the absence of cDNA. Expression data were generated from amplification reactions with samples and controls run in triplicate and performed on different cDNA samples reverse transcribed from RNA prepared from independent culture assays. Optical data obtained by real-time PCR were analyzed using the Thermal Cycler Dice Real-Time System II Software Version 5.11 (Takara Bio Inc., Shiga, Japan). The expressions of housekeeping genes such as β-actin were analyzed. All primers were synthesized commercially (Sigma-Aldrich, Saint Louis, Missouri). The sequences of the primer pairs are detailed in **▶** Table 2 .

**Table 2:** Primers and Conditions Used for Real-Time RT-PCR

Gene	Forward primer (5′-3′)	Reverse primer (5′-3′)
β-actin	AGCCTCGCCTTTGCCGATCC	TTGCACATGCCGGAGCCGTT
lntegrin β4	CGCCGTCTGGTAAACATC	AGTAGCTTCACCTGCAACTC
TNF-α	TTCTCCTTCCTGATCGTGGC	TCGAGAAGATGATCTGACTGCC

Abbreviations: RT-PCRs, reverse transcription polymerase chain reactions; TNF-α, tumor necrosis factor-α.

### Statistical Analysis

Nonparametric relative expression data were analyzed using the Kruskal–Wallis test to compare leukocyte count, absorbance of WST-1, percent of the area of wound healing, and log-transformed mRNA expression levels among PC, NC, PRGF, and PRP. All statistical analyses were performed using EZR (Saitama Medical Center, Jichi Medical University, Saitama, Japan), a graphical user interface for R ( www.r-project.org ; The R Foundation for Statistical Computing, Vienna, Austria). 25 *p* -values <0.05 were considered statistically significant.

## Results

### Cytological Feature and Leukocyte Count of PRGF and PRP

**Fig. 1:**
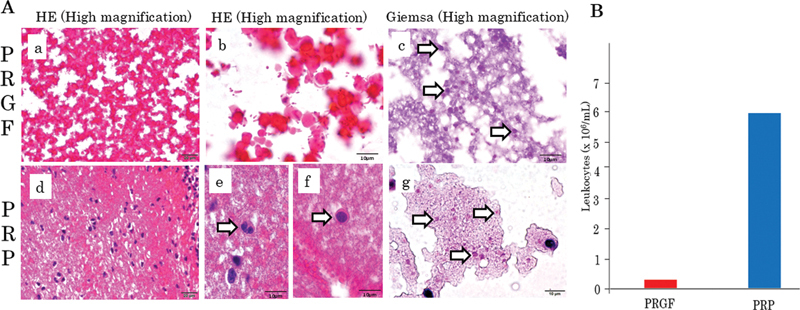
( **A** ) Representative blood components of PRGF and PRP. Platelets are shown in PRGF (A [a–c], arrows). PRP contains platelets (A[g], arrows), including leukocytes such as neutrophils (A [e], arrow) and lymphocytes (A [f], arrow). ( **B** ) Leukocyte count of PRGF and PRP. Two different plasma preparations as PRGF and PRP of the three donors. Although not significant, the leukocyte concertation in PRP tends to be higher than that in PRGF ( *p* = 0.089). The data are expressed as a median. PRGF, plasma rich in growth factors; PRP, platelet-rich plasma.

At first, we qualitatively examined the blood components of PRGF and PRP using the cell block method and conventional Hematoxylin-Eosin and Giemsa staining. The Hematoxylin-Eosin section presented the representative blood components of the PRGF and PRP groups ( **▶** Fig. 1A [a–f] ). The PRGF group contained only platelets without leukocytes ( **▶** Fig. 1A [a, b] ). In contrast, there were a lot of leukocytes in the PRP group ( **▶** Fig. 1A [d–f] ). The leucocytes were composed of many neutrophils ( **▶** Fig. 1A [e] ) and a few lymphocytes ( **▶** Fig. 1A [f] ). Additionally, the Giemsa-stained section exhibited a large number of platelets, which appeared red-orange in both PRGF ( **▶** Fig. 1A [c] ) and PRP ( **▶** Fig. 1A [g] ) groups. We quantitatively examined the blood components, such as the leukocytes, of the PRGF and PRP groups. Although not significant, the leukocyte concentration in the PRP group tended to be higher than that in PRGF one ( *p* = 0.089; **▶** Fig. 1B ; **▶** Table 1 ).

### Cell Proliferation

**Fig. 2:**
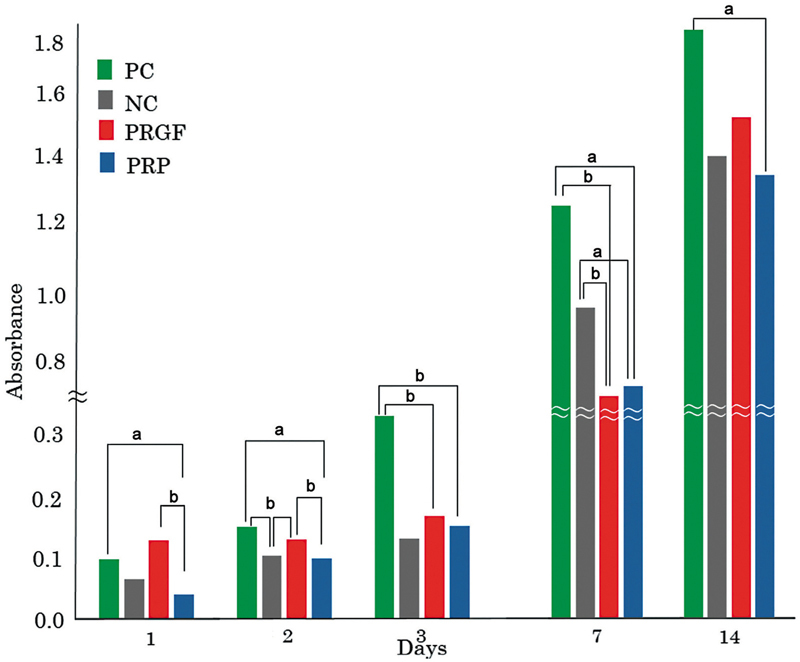
Cell proliferation. Quantification of gingival epithelial cells' proliferation on PC, NC, PRGF, and PRP. There are statistically significant differences in cell proliferation between PRGF and PRP in comparison to the PC and NC on days 1 and 2 ( ^a^ *p* < 0.05, ^b^ *p* < 0.001). All data are expressed as a median. NC, negative control; PC, positive control; PRGF, plasma rich in growth factors; PRP, platelet-rich plasma.

The absorbance was measured after adding WST-1 to HGEPp. The WST-1 test showed that the absorbance of PRGF was significantly higher than that of PRP on day 1 ( *p* = 0.008) and day 2 ( *p* = 0.003). On day 1, the absorbance in the PRP group was significantly lower than that in the PC ( *p* = 0.009). On day 2, the absorbance was significantly higher in the PRGF group than in the NC ( *p* = 0.036). The PRP group displayed a significantly lower absorbance than the PC on day 2 ( *p* = 0.012). On days 3, 7, and 14, there was no significant difference in absorbance between the PRGF and PRP groups ( **▶** Fig. 2 ).

### Wound Healing Assay

**Fig. 3:**
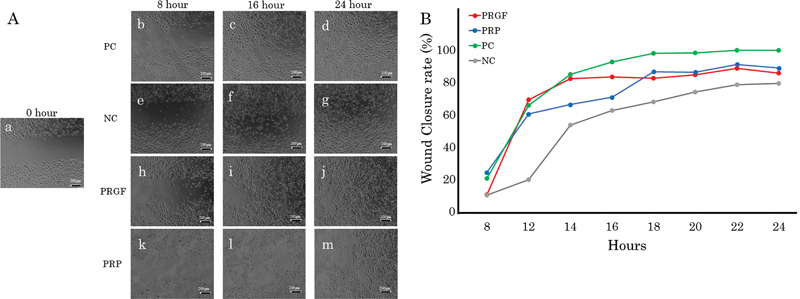
Wound healing assay on gingival epithelial cells. ( **A** ) Representative image of wound healing over time under different treatments (8, 16, 24 hours) as PC ( **B–D** ), NC ( **E–G** ), PRGF ( **H–J** ), and PRP ( **K–M** ). At hour 0, the phase contrast microscope exhibits a diameter of 500 µm intracellular space ( **A** ). ( **B** ) Median of the wound closure rate of gingival epithelial cells after treatment with the different cell culture mediums belonging to the PC, NC, PRGF, and PRP during the 8- to 24-hour follow-up. There is no significant difference in the wound closure rate shown by the PRGF and PRP groups in comparison to the PC and NC at each interval ( *p* > 0.05). NC, negative control; PC, positive control; PRGF, plasma rich in growth factors; PRP, platelet-rich plasma.

We examined the wound closure rate of HGEPp under the influence of PRGF and PRP, PC, and NC. **▶** Fig. 3A shows the state of wound closure affected by each cell culture medium during the 8- to 24-hour follow-up. The chronological photography on 0 hour exhibited a diameter of 500 µm intracellular space immediately after removing the cell culture insert. **▶** Fig. 3B shows the cell closure ratio of the gingival epithelial cells under the different cell culture mediums. The statistical results showed no significant difference in the wound closure rate of the PRGF and PRP groups, by comparison with each of respective control groups at each observation time ( *p* > 0.05).

### Quantitative RT-PCR

**Fig. 4:**
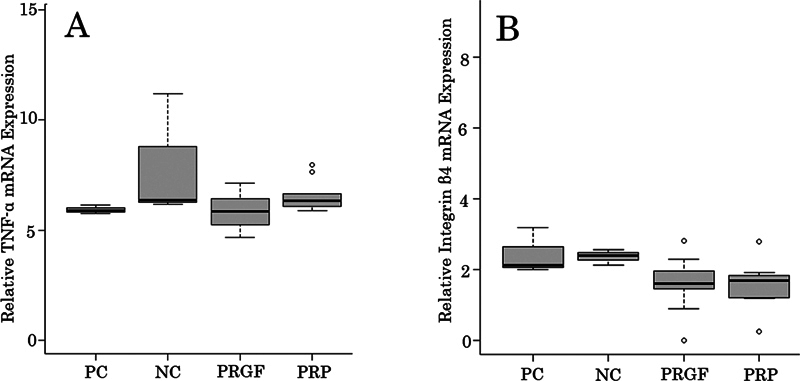
TNF-α (A) and integrin β4 (B) mRNA expression of gingival epithelial cells. Gingival epithelial cells show no significant gene expression levels such as TNF-α and integrin β4 between PRGF, PRP, PC, and NC ( *p* > 0.05). All data are expressed as a median. NC, negative control; PC, positive control; PRGF, plasma rich in growth factors; PRP, platelet-rich plasma; TNF-α, tumor necrosis factor-α.

The mRNA levels of both tumor necrosis factor-α (TNF-α) and integrin β4 were not significant in the comparison between the PRGF and PRP groups and the positive and NCs ( *p* > 0.05; **▶** Fig. 4A, B ). The expression levels of each gene were normalized by the quantitative value of β-actin.

## Discussion

In our research, we hypothesized that PRGF containing few leukocytes enhances the proliferation and healing of gingival epithelial cells in comparison to leukocyte-rich PRP in vitro. Our results indicated that PRGF tended to have fewer white blood cells than PRP. In addition, both PRPG and PRP had large numbers of platelets. The cell proliferation was significantly higher in the PRGF group than in the PRP group on days 1 and 2. However, there was no significant difference in the wound closure rate between both groups in comparison to their respective control groups. Moreover, a quantitative RT-PCR indicated no significant difference in the gene expressions of TNF-α and integrin β4 in comparison to each of their respective control groups.

Previous in vitro studies of PRGF have limited their focus to periodontal mesenchymal cells, such as gingival fibroblast and osteoblast. 21 However, the influence of PRGF on gingival epithelium remains poorly understood. To the best of our knowledge, this is the first study to clarify that PRGF affects epithelial cells (especially gingival epithelial cells) in terms of proliferation, wound closure rate, and the associated gene expression.

In the early days, PRGF was found to be responsible for cell proliferation as opposed to PRP. In contrast, the quantitative RT-PCR indicated no significant difference between either group in the gene expression of TNF-α, which is understood to suppress cell proliferation, relative to their respective control groups. Previous research revealed that the synthesis of TNF-α is significantly increased when 10 ng/mL IL-8 is exposed to gingival epithelial cells. 26 However, the IL-8 content in PRGF is 0.1 ng/µL, and that in even PRP is only 3.5 ng/µL. 19 In addition, less than 80 ng/mL of TNF-α does not affect cell viability. 26 The concentration of IL-8 derived from PRP may thus be insufficient for the production of TNF-α not to decrease cell viability.

The significant difference in proliferation between PRGF and PRP can be attributed to the concentration of IL-1β. The IL-1β content in PRP is significantly higher than that in PRGF. 15 A previous comprehensive analysis of genes with immortalized human gingival epithelial cells treated with IL-1β showed upregulation of ornithine decarboxylase contributing to apoptosis, while downregulation of classical cell cycle promoting genes such as cyclin B. 27 Given this context, PRGF is likely to increase proliferation due to the influence of IL-8.

Relative to the respective control groups, there was no significant difference between either groups in terms of the wound closure rate and the gene expression of TNF-α of gingival epithelial cells. Previous literature has reported that TNF-α induces regulators of the actin cytoskeleton in epidermal keratinocytes, increasing their motility. 28 In addition, gingival epithelial cells promote migration by increasing actin production. 29 For this reason, the expression levels of TNF-α mRNA in gingival keratinocytes may not cause changes to the actin cytoskeleton, contributing to no alteration in motility.

A wounding healing model with Diabetic rats and in vitro research revealed that macrophages releasing TNF-α plays an important role in re-epithelialization, through cell migration rather than cell proliferation. 30 A study with normal mice and a TNF-α neutralizing antibody clarified that the physiological low level of TNF-α is involved in the process of wound healing in the skin. 31 In our study, the gene expression levels of TNF-α in the PRGF and PRP groups were not different from those in the control group. This result suggests that they did not alter epithelial cell migration, causing no significant changes in the rate of wound closure.

The lack of any significant difference in wound closure rate between PRGF and PRP can be attributed to the concentration of EGF and integrin β4. Several previous studies have indicated that 5,000 pg/mL and 10,000 pg/mL of EGF cause an increase in the expression of integrin β4 of mouse 32 and humans 33 keratinocytes, respectively, whereas the administration of EGF immediately after the scratching of cells significantly promoted the migration of mouse 32 keratinocytes. However, the concentration of EGF in PRP and PRGF were only 279 pg/mL and 489 pg/mL, respectively. 34 In other words, these concentrations are lower than the ones mentioned above. As discussed so far, these lower concentrations of EGF in PRGF and PRP may cause no significant difference in the integrin β4 mRNA expression levels, thus contributing negligibly to the migration speed of gingival epithelial cells.

Research with scratch-wounded airway epithelial cells indicated that an integrin-β4 influences actin reorganization, thereby accelerating cell proliferation and the wound closure rate. 35 In contrast, actin cytoskeleton regulates the movements of α6β4 integrin. 36 Therefore, integrin β4 and actin have a bidirectional relationship and are involved in cell migration. This explains why there was no difference in integrin β4 gene expression and the wound closure rate between the PRGF and PRP groups.

PRGF promoted cell proliferation more effectively than PRP on days 1 and 2, but no significant differences in wound closure rates were observed. It has been reported that changes in wound area occur when the migration and proliferation of mouse keratinocytes are inhibited separately in vivo. 37 Following the inhibition of migration, wound repair is directly delayed. In contrast, following the inhibition of proliferation, the tissue compensates by increasing the overall size of the repair region to cause no significant delays in wound repair. 37 Therefore, the low proliferation of L-PRP could have been compensated for by the increased tissue size of the repair cells. In other words, the proliferative advantage of PRGF did not contribute to improving wound closure outcomes compared with L-PRP.

PRGF and PRF induce the antimicrobial peptides β-defensin-2 in keratinocytes. 38 In addition, PRGF treatment of keratinocytes caused an increase in the expression of the psoriasin gene and protein that were mediated by EGFR and IL-6R. 38 Moreover, PRF induced a significant expression of the psoriasin gene and protein when applied to skin wounds. 38 Therefore, PRGF and PRF can induce further antimicrobial peptides in keratinocytes, demonstrating the importance of their effect on growth factors and interleukin, their positive impact on the epithelial barrier, and their usefulness for the wound healing process.

This study has certain limitations. First, PRGF and PRP were obtained from blood samples from only three Mongoloid male volunteers in their 20s and 30s. In contrast, the primary cultured gingival epithelial cells used in our experiment were commercially obtained from three Caucasoid females in their 20s. Therefore, gender, age, and individual differences were not accounted for in our experiments. The experiment thus did not reflect clinical practice for applying platelet concentrates. In subsequent research, we aim to receive both blood and cells from donors. Second, we did not quantify the concentration of the interleukins and growth factors derived from leukocytes and platelets. The concentrations of interleukins and growth factors used in the discussion section are based on previous research on PRGF and PRP. 15 19 34 In subsequent research, we aim to quantify interleukins and growth factors in PRP and PRGF. Then, we will examine the relationship among them quantitatively in terms of the absorbance with WST-1 and percent of wound healing.

## Conclusion

In summary, our research indicated that PRGF can promote the proliferation of gingival epithelium more than PRP, contributing to the healing of periodontal tissue wounds. TNF-α and integrin β4 mRNA expression may not be significantly implicated in wound closure with gingival epithelial cells, affected by PRGF and PRP.

## References

[1] Werner S Krieg T Smola H Keratinocyte-fibroblast interactions in wound healing J Invest Dermatol 2007 127 05 998 1008 17435785 10.1038/sj.jid.5700786

[2] Presland R B Jurevic R J Making sense of the epithelial barrier: what molecular biology and genetics tell us about the functions of oral mucosal and epidermal tissues J Dent Educ 2002 66 04 564 574 12014572

[3] Tsukita S Furuse M Claudin-based barrier in simple and stratified cellular sheets Curr Opin Cell Biol 2002 14 05 531 536 12231346 10.1016/s0955-0674(02)00362-9

[4] Hosokawa I Hosokawa Y Komatsuzawa H Innate immune peptide LL-37 displays distinct expression pattern from beta-defensins in inflamed gingival tissue Clin Exp Immunol 2006 146 02 218 225 17034573 10.1111/j.1365-2249.2006.03200.x PMC1942065

[5] Kimball J R Nittayananta W Klausner M Chung W O Dale B A Antimicrobial barrier of an in vitro oral epithelial model Arch Oral Biol 2006 51 09 775 783 16815238 10.1016/j.archoralbio.2006.05.007 PMC2376809

[6] Guo S Dipietro L A Factors affecting wound healing J Dent Res 2010 89 03 219 229 20139336 10.1177/0022034509359125 PMC2903966

[7] Bakhshandeh B Zarrintaj P Oftadeh M O Tissue engineering; strategies, tissues, and biomaterials Biotechnol Genet Eng Rev 2017 33 02 144 172 29385962 10.1080/02648725.2018.1430464

[8] Cecerska-Heryć E Goszka M Serwin N Applications of the regenerative capacity of platelets in modern medicine Cytokine Growth Factor Rev 2022 64 84 94 34924312 10.1016/j.cytogfr.2021.11.003

[9] Slater M Patava J Kingham K Mason R S Involvement of platelets in stimulating osteogenic activity J Orthop Res 1995 13 05 655 663 7472743 10.1002/jor.1100130504

[10] Marx R E Carlson E R Eichstaedt R M Schimmele S R Strauss J E Georgeff K R Platelet-rich plasma: Growth factor enhancement for bone grafts Oral Surg Oral Med Oral Pathol Oral Radiol Endod 1998 85 06 638 646 9638695 10.1016/s1079-2104(98)90029-4

[11] Hu Z Xv H Feng A Wang S Han X Efficacy and safety of platelet-rich plasma for pressure ulcers: a systematic review and meta-analysis of randomized controlled trials Int J Low Extrem Wounds 2024 15347346241227001 10.1177/15347346241227001 38239009

[12] Pallua N Wolter T Markowicz M Platelet-rich plasma in burns Burns 2010 36 01 4 8 19541423 10.1016/j.burns.2009.05.002

[13] Charrier J B Monteil J P Albert S Collon S Bobin S Dohan Ehrenfest D M Relevance of Choukroun's platelet-rich fibrin (PRF) and SMAS flap in primary reconstruction after superficial or subtotal parotidectomy in patients with focal pleiomorphic adenoma: a new technique Rev Laryngol Otol Rhinol (Bord) 2008 129(4–5):313 318 19408518

[14] Weibrich G Kleis W KG Hitzler W E Hafner G Comparison of the platelet concentrate collection system with the plasma-rich-in-growth-factors kit to produce platelet-rich plasma: a technical report Int J Oral Maxillofac Implants 2005 20 01 118 123 15747683

[15] Masuki H Okudera T Watanebe T Growth factor and pro-inflammatory cytokine contents in platelet-rich plasma (PRP), plasma rich in growth factors (PRGF), advanced platelet-rich fibrin (A-PRF), and concentrated growth factors (CGF) Int J Implant Dent 2016 2 01 19 27747711 10.1186/s40729-016-0052-4 PMC5005757

[16] Dohan Ehrenfest D M Rasmusson L Albrektsson T Classification of platelet concentrates: from pure platelet-rich plasma (P-PRP) to leucocyte- and platelet-rich fibrin (L-PRF) Trends Biotechnol 2009 27 03 158 167 19187989 10.1016/j.tibtech.2008.11.009

[17] Orcajo B Muruzabal F Isasmendi M C The use of plasma rich in growth factors (PRGF-Endoret) in the treatment of a severe mal perforant ulcer in the foot of a person with diabetes Diabetes Res Clin Pract 2011 93 02 e65 e67 21546112 10.1016/j.diabres.2011.04.008

[18] Anitua E Muruzabal F De la Fuente M Merayo-Lloves J Orive G Effects of heat-treatment on plasma rich in growth factors-derived autologous eye drop Exp Eye Res 2014 119 27 34 24345372 10.1016/j.exer.2013.12.005

[19] Anitua E Zalduendo M Troya M Padilla S Orive G Leukocyte inclusion within a platelet rich plasma-derived fibrin scaffold stimulates a more pro-inflammatory environment and alters fibrin properties PLoS One 2015 10 03 e0121713 25823008 10.1371/journal.pone.0121713 PMC4379078

[20] Yadav A Ramasamy T S Lin S C Autologous platelet-rich growth factor reduces M1 macrophages and modulates inflammatory microenvironments to promote sciatic nerve regeneration Biomedicines 2022 10 08 1991 36009539 10.3390/biomedicines10081991 PMC9406033

[21] Anitua E Zalduendo M Troya M Tierno R Alkhraisat M H The inclusion of leukocytes into platelet rich plasma reduces scaffold stability and hinders extracellular matrix remodelling Ann Anat 2022 240 151853 34767933 10.1016/j.aanat.2021.151853

[22] Talebi Ardakani M R Meimandi M Shaker R Golmohammadi S The effect of platelet-rich fibrin (PRF), plasma rich in growth factors (PRGF), and enamel matrix proteins (Emdogain) on migration of human gingival fibroblasts J Dent (Shiraz) 2019 20 04 232 239 31875169 10.30476/DENTJODS.2019.44917 PMC6890812

[23] Zhang S W Chen W Lu X F An efficient and user-friendly method for cytohistological analysis of organoids J Tissue Eng Regen Med 2021 15 11 1012 1022 34555270 10.1002/term.3248

[24] Meier L Carlson R Neßler J Tipold A Stability of canine and feline cerebrospinal fluid samples regarding total cell count and cell populations stored in “TransFix®/EDTA CSF sample storage tubes” BMC Vet Res 2020 16 01 487 33334339 10.1186/s12917-020-02698-5 PMC7745459

[25] Kanda Y Investigation of the freely available easy-to-use software ‘EZR’ for medical statistics Bone Marrow Transplant 2013 48 03 452 458 23208313 10.1038/bmt.2012.244 PMC3590441

[26] Basso F G Pansani T N Turrioni A PS Soares D G de Souza Costa C A Hebling J Tumor necrosis factor-α and interleukin (IL)-1β, IL-6, and IL-8 impair in vitro migration and induce apoptosis of gingival fibroblasts and epithelial cells, delaying wound healing J Periodontol 2016 87 08 990 996 27063996 10.1902/jop.2016.150713

[27] Steinberg T Dannewitz B Tomakidi P Analysis of interleukin-1beta-modulated mRNA gene transcription in human gingival keratinocytes by epithelia-specific cDNA microarrays J Periodontal Res 2006 41 05 426 446 16953820 10.1111/j.1600-0765.2006.00884.x

[28] Banno T Gazel A Blumenberg M Effects of tumor necrosis factor-alpha (TNF alpha) in epidermal keratinocytes revealed using global transcriptional profiling J Biol Chem 2004 279 31 32633 32642 15145954 10.1074/jbc.M400642200

[29] Rouabhia M Rouabhia D Park H J Giasson L Zhang Z Effect of soft foods on primary human gingival epithelial cell growth and the wound healing process Food Res Int 2017 100(Pt 1):433 441 28873706 10.1016/j.foodres.2017.07.041

[30] Scott K A Arnott C H Robinson S C TNF-alpha regulates epithelial expression of MMP-9 and integrin alphavbeta6 during tumour promotion. A role for TNF-alpha in keratinocyte migration? Oncogene 2004 23 41 6954 6966 15273742 10.1038/sj.onc.1207915

[31] Ritsu M Kawakami K Kanno E Critical role of tumor necrosis factor-α in the early process of wound healing in skin J Dermatol Dermatol Surg 2017 21 01 14 19

[32] Carey T E Laurikainen L Nair T S Regulation of expression and phosphorylation of A9/alpha 6 beta 4 integrin in normal and neoplastic keratinocytes J Natl Cancer Inst Monogr 1992 13 75 86 1389698

[33] Miyazaki A Ohkubo T Hatta M Ishikawa H Yamazaki J Integrin α6β4 and TRPV1 channel coordinately regulate directional keratinocyte migration Biochem Biophys Res Commun 2015 458 01 161 167 25637531 10.1016/j.bbrc.2015.01.086

[34] Freire V Andollo N Etxebarria J Durán J A Morales M C In vitro effects of three blood derivatives on human corneal epithelial cells Invest Ophthalmol Vis Sci 2012 53 09 5571 5578 22786903 10.1167/iovs.11-7340

[35] Tan M L Huang W J Wang Y Integrin-β4 regulates the dynamic changes of phenotypic characteristics in association with epithelial-mesenchymal transition (EMT) and RhoA activity in airway epithelial cells during injury and repair Int J Biol Sci 2022 18 03 1254 1270 35173551 10.7150/ijbs.65174 PMC8771845

[36] Hamill K J Hiroyasu S Colburn Z T Alpha actinin-1 regulates cell-matrix adhesion organization in keratinocytes: consequences for skin cell motility J Invest Dermatol 2015 135 04 1043 1052 25431851 10.1038/jid.2014.505 PMC4366307

[37] Park S Gonzalez D G Guirao B Tissue-scale coordination of cellular behaviour promotes epidermal wound repair in live mice Nat Cell Biol 2017 19 02 155 163 28248302 10.1038/ncb3472 PMC5581297

[38] Bayer A Lammel J Rademacher F Platelet-released growth factors induce the antimicrobial peptide human beta-defensin-2 in primary keratinocytes Exp Dermatol 2016 25 06 460 465 26843467 10.1111/exd.12966

